# Fussy Eating among Children and Their Parents: Associations in Parent-Child Dyads, in a Sample of Children with and without Neurodevelopmental Disorders

**DOI:** 10.3390/nu13072196

**Published:** 2021-06-25

**Authors:** Sigrun Thorsteinsdottir, Annemarie Olsen, Anna S. Olafsdottir

**Affiliations:** 1 Faculty of Health Promotion, Sport and Leisure Studies, School of Education, University of Iceland, 105 Reykjavík, Iceland; annaso@hi.is; 2 Department of Food Science, University of Copenhagen, 1958 Copenhagen, Denmark; ano@food.ku.dk

**Keywords:** fussy eating, eating behaviors, neurodevelopmental disorders, ADHD, autism spectrum disorder, parent-child dyads

## Abstract

Parents are important agents in shaping children’s eating habits. However, the associations between children’s and parents’ eating behaviors are complex and may be convoluted for various reasons, such as parenting feeding styles, stressful mealtimes, and children’s neurodevelopmental disorders (ND), such as Autism Spectrum Disorder (ASD) and Attention-Deficit/Hyperactivity Disorder (ADHD). The purpose of this study was to analyze associations between parents and their children’s fussy eating, in a cross-sectional sample of children, with and without ND. Ninety-seven parents answered screening questionnaires prior to an intervention study. Associations were investigated using two-way ANOVAs and chi-square analyses. Overall, children with ND accepted fewer food items and consumed unhealthier foods more frequently than children without ND. Fussy eating parents had children who accepted fewer food items and consumed unhealthier foods more frequently than children whose parents were not fussy eaters. Interaction effects were not significant. A higher proportion of fussy eating parents, than non-fussy eating parents, had children who had difficulties with combined foods and hidden ingredients. The findings highlight the need for further investigation into the relationships between parents’ influence on their children’s eating behavior and food consumption, as well as possible reciprocal impacts.

## 1. Introduction

A nutritious and varied diet is important for overall health and well-being and studies have shown that a diet rich in plant foods such as fruit, vegetables, legumes, pulses, nuts, seeds, and wholegrains, is associated with a lower risk of all-cause and cardiovascular disease mortality [1,2]. However, daily intake of fruit and vegetables is well below the recommendations of five portions a day for children [3,4] and adults [5]. Children’s preferences for, and consumption of, fruit and vegetables and less healthy options such as sugary snacks have been shown to persist into adulthood, highlighting the importance of promoting healthy eating habits from an early age [6,7]. Parents are important agents in molding eating habits, however, the associations between children’s and parents’ eating behaviors are complex and may be convoluted for various reasons, such as parenting feeding style, stressful mealtimes [8,9,10], and children’s neurodevelopmental disorders (ND) such as Autism Spectrum Disorder (ASD) and Attention-Deficit/Hyperactivity Disorder (ADHD). 

Food rejection is common among young children, where 20–30% of children between the ages of two and six may have difficult eating behaviors and stringent food preferences [11,12]. Restricted eating behaviors may include *food neophobia*, the rejection of new or unknown foods [13,14], and *fussy* or *picky* eating, the rejection of a large proportion of novel and familiar foods, leading to a routine diet where the individual’s intake of foods is especially low in variety [13,15]. Furthermore, disrupted eating behaviors may result in adverse health-related outcomes in the long-term [11,13,16]. Since fussy eating includes food neophobia, both terms are used here as fussy eating. 

Although the symptoms of fussy eating in later childhood and beyond are diverse across children, many share similar characteristics when faced with novel or unliked foods. These include fear, anxiety, mistrust, and disgust [13,17]. These problems may be exacerbated for children who are not typically developing (TD), including children with ND [18,19,20]. Fussy eating tends to peak in early childhood and then reduce [21] but less so for children with ND [22]. Rates of fussy eating may reach 80% in children with ASD [23,24] and around 40% in children with ADHD [24,25,26]. Furthermore, children with ASD may be on specialized diets—for example alternative diets, eliminating dairy or gluten—in an attempt to reduce behavioral symptoms of ASD [27,28]. One multi-site study reported that 20% of preschool children with ASD had tried a gluten-free diet but the effectiveness of the eliminated food on behavioral symptoms was still uncertain [29]. A recent randomized, controlled, single-blinded trial found no differences between groups comparing gluten-free diet and gluten-containing diet, in measures including symptoms of ASD and maladaptive behaviors [28]. Eliminating certain foods from children’s diets may reduce gastrointestinal problems in some instances [30], however, this may be irrespective of children’s ND-status and may negatively affect children’s diet and food choices.

Young children commonly refuse to taste novel foods or foods that may be mushy, with tough textures and “bits” in them, as well as dishes with “hidden” ingredients such as lasagna and casseroles [31,32]. Vegetables may also prove particularly challenging as they have bitter taste profiles which may be difficult for fussy eaters, especially children with ND [33,34]. Research on children with ND has shown sensory sensitivities towards textures and tastes, especially in fibrous foods such as fruit and vegetables [26,35,36]. This increased sensitivity to sensory experiences, such as taste, smell, and touch, are characteristics that are also highly associated with fussy eating [37]. This may be one of the reasons fussy eaters, particularly those with ND, often have diets consisting of foods that are bland in color and lacking in textures and taste, as well as being low in nutrient density [24,35,36].

Difficult eating behaviors are influenced by many factors and may be very worrisome for parents, adding to the stress of raising children with ND [20,38,39]. Furthermore, parents’ and children’s difficult eating behaviors may be reciprocal, leading to parental stress [40], especially in the challenges encountered when trying to expand children’s diet towards healthier options [9,41]. Research shows that children’s food preferences and habits can persist into adulthood [6,7] and children may resemble members of their families in their food preferences [42]. Young adults and their parents have also been shown to resemble each other in food related behaviors, with positive associations between parents’ and children’s self-reported fussy eating [43]. In one study, mothers who were fussy eaters themselves were less likely to offer healthy foods to their children [44], and, in another study, maternal preferences corresponded with child preferences [45]. Furthermore, another study showed that parental fruit and vegetables consumption was the strongest predictor of their children’s intake [46]. However, parents can also exert a very positive influence on food preferences and children’s eating and mealtime behaviors, especially when combined with family-based interventions [9,13,17,47]. However, none of these studies have examined associations between eating behaviors of parents, and children with and without ND.

The purpose of this study was to analyze the association between parents and their children’s fussy eating. In particular, to investigate fussy eating and dietary behaviors as well as food acceptance and frequency of consumption in a sample of children, with and without ND, and the associations with their parents’ fussy eating.

## 2. Materials and Methods

Data presented in the current study is based on a longitudinal, randomized controlled study of a taste education intervention (for a detailed description of the study see [48]). We used cross-sectional screening data for the children which initially was gathered for selecting participants for the intervention, while information on parents was gathered at baseline, after being invited to take part in the intervention.

### 2.1. Measures

#### 2.1.1. Parents’ and Children’s Eating Behaviors

Demographic information (children’s age and sex, as well as parental education, occupation, and marital status) was obtained from the screening questionnaires developed by the researchers. The Adult Picky Eating Questionnaire by Kauer et al. [48] was used as a basis for a questionnaire designed to obtain information on parents’ eating behaviors. The list comprised 41 “True” or “False” statements such as: “I am a fussy eater”, “I try not to let different types of foods touch on my plate”. The questionnaire includes the following categories: Other eating behaviors; Narrow range; Neophobia; Sensory rejection: Taste; Sensory rejection: Texture; Sensory rejection: Appearance; Contact or mixing; Ritualization/repetition; Interest in food/social eating. Cronbach’s alpha was 0.63 in our sample which is an acceptable level of internal consistency. Picky Eating in Children (modified from Adult Picky Eating) was then used as a basis for parental reporting on their children’s eating behaviors: The list also comprised “True” or “False” statements on children’s fussy eating and food behaviors such as: “My child always rejects foods that have touched on the plate”. Cronbach’s alpha, in our sample, was 0.73 which indicates a good level of internal consistency.

Parents’ fussy eating status was based on their eating behavior responses which were split into two groups, based on 14 statements that were chosen from the list of 41 “Yes/No” statements from the Adult Picky Eating Questionnaire [48] (Table 1). The statements were independently selected by two researchers to represent fussy eating. The researchers agreed unanimously on the selection. Those parents who were classified into Fussy eaters, responded with “Yes” to all 14 questions. Other statements from the list included questions on food allergies, religious reasons, diets, or healthy eating which were not used. After the selection, 38.1% of the parents were considered fussy eaters (Table 2).

#### 2.1.2. Children’s Food Consumption: Acceptance

Children’s food acceptance and variety were assessed using a parent-reported dietary intake questionnaire of 58 plant-based foods (Fruit index: Pineapple, oranges, green apples, red apples, bananas, pomegranates, kiwis, satsumas, mangoes, watermelons, honeydew melons, pears, green grapes, and blue grapes. Vegetables, kale, and salads index: Cucumbers, cauliflower, eggplant, carrots, kohlrabi, zucchini, yellow onions, red onions, avocadoes, olives, leeks, red/yellow peppers, green peppers, radishes, beets, turnips, celery stalks, broccoli, mushrooms, tomatoes, leafy greens, kale, baked kale, white cabbage, iceberg, Chinese cabbage, rucola, red cabbage, and spinach. Nuts and dried fruits index: hazelnuts, peanuts, cashew nuts, desiccated coconut, almonds, dried dates, raisins, dried cranberries, and dried apricots. Seeds index: Poppy, pumpkin, sesame, sunflower seeds and linseeds). The questionnaire was grouped into four indices: Fruit index; Vegetables, kale, and salads index; Nuts and dried fruits index; and Seeds index. For each participant, the number of accepted food items for each index was tallied into a food acceptance and variety score. The response choices included “Yes”, “Sometimes” (recoded into “Yes”), and “No”, “Not sure”, “Not familiar with” (recoded into “No”). For the chi-square analyses the “Yes” responses were used for comparing percentages of parents’ and children’s food acceptance.

#### 2.1.3. Measures of Food Frequency

Parents were asked to assess how often their children consumed a particular food by answering a parent-reported dietary frequency questionnaire. Each question included 8 response options. To facilitate analyses, the scale was adjusted to “times per week“ on an ordinal scale from 0 to 21. The answering options were: “Never” = 0, “Less than once per week” = 0.5, “Once per week” = 1, “2–3 times per week” = 2.5, “4–5 times per week” = 4.5, “6–7 times per week” = 6.5, “Twice per day” = 14, “3 times per day or more” = 21.

### 2.2. Participants

Of potentially 190 eligible parent-child dyads who completed the screening questionnaire for consent and eligibility, 95 (50.0%) agreed to participate and 81 completed the full intervention. While 95 participants agreed to participate (Table 2), the data for the current study consisted of 97 parent-child dyads. Of those 97 parents, two children had both parents (divorced and living in separate homes) fill out questionnaires. The screening/baseline data was collected over one year, starting in 2018. All questionnaires were administered and stored online using Qualtrics software (Qualtrics, Provo, UT, USA).

Parents of 8–12-year-old fussy eaters with and without ND were invited to participate in the study. Children who were seven years old at the time of screening, up to 12 years old, were accepted to participate. Participants were invited through communications with social media, through email lists in partnership with the Icelandic ASD and ADHD societies, and via adverts on a website dedicated to the study. To ensure validation of children’s ADHD and ASD diagnoses, all children were required to have been diagnosed by one of the three major Icelandic diagnostic centers which all use standardized diagnostic instruments and protocols. All participants were Icelandic and the majority was living in the capital and surrounding regions. The majority had an education at a university level and most were in full-time occupation. The majority of parents were also married. Inclusion criteria encompassed fussy eating in children and Icelandic speaking parents and children. There were no requirements for parents to be fussy eaters. The children needed to have sufficient dexterity to feed themselves without difficulty. Questions on children’s fussy eating, language, and dexterity were included in the screening questionnaire. All children attended mainstream schooling. Since the focus was on food acceptance and frequency and not amount, children were included regardless of use of certain medications such as methylphenidate, which may affect appetite. All children with ADHD, apart from two, were on methylphenidate medication.

Those wishing to participate were asked to provide informed consent by selecting the applicable option at the end of the information sheet. Parents were informed that the intervention would not interfere with other services the child was receiving elsewhere or exclude them from receiving them. Participants were not financially compensated for their participation in the study.

### 2.3. Statistical Analysis

Data were analyzed using IBM^®^ SPSS^®^ Statistics 26.0 (IBM Corp. Armonk, NY, USA, 2019). Descriptive statistics were calculated and presented as n (%). Graphs were created using R version 4.0.3 [49]. A two-way ANOVA was conducted to examine the effects of parents’ fussy eating and children’s ND-status on food acceptance scores, on the four food indices (Fruit index, Vegetables, kale and salads index, Nuts, and dried fruits index, and Seeds index). A two-way ANOVA was also used to examine the effects of parents’ fussy eating and children’s ND-status on food frequency scores on various food items. Residual analysis was performed to test for two-way ANOVA assumptions. Outliers were assessed by inspecting boxplots, normality was assessed using Shapiro-Wilk’s normality test, and homogeneity of variances was assessed by Levene’s test. There were no extreme outliers, residuals were normally distributed (*p* > 0.05) and there was homogeneity of variances. Cases with missing data were excluded listwise.

Associations between parents’ fussy eating status and children’s food behaviors were analyzed using chi-square tests. The 30 questions associated with children’s fussy eating are based on the conceptual grouping of the original questionnaire by Kauer et al. [48].

Although multiple hypotheses tests were conducted, unadjusted alphas were reported [50,51].

## 3. Results

Characteristics of parents and children in the study are presented in Table 2. Just over half of the children were diagnosed with ND (50.5%). The mean age of the children was 9.9 years (SD 1.51; range 7–13 years-old) and more than half of the participants were female (56.8%). The parents consisted mostly of mothers (94.8%). The proportion of parents who were regarded as being fussy eaters, was 38.1%.

### 3.1. Children’s Food Acceptance Based on Parents’ Fussy Eating and Children’s ND-Status

A two-way ANOVA was conducted to examine the effects of parents’ fussy eating status and children’s ND-status on food acceptance scores (Table 3). The score measured how many foods the children accepted within a particular food index. The trend pointed towards higher mean acceptance scores on all food indices for non-fussy eating parents, and lower acceptance scores for fussy eating parents. The trend also indicated lower means in general for children with ND than without ND.

**Figure 1:**
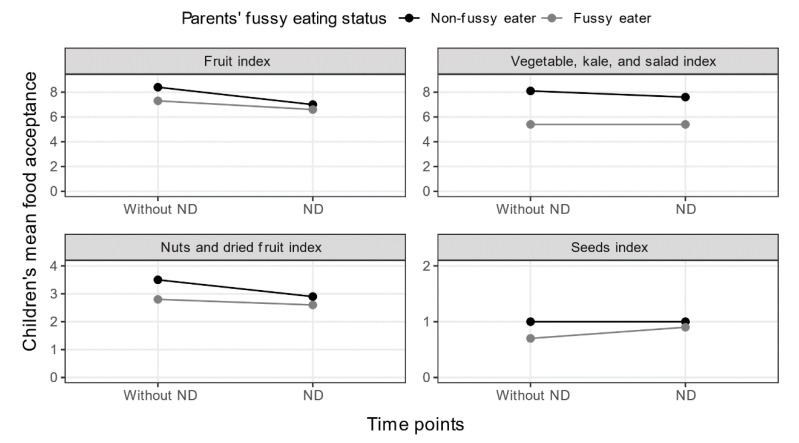
Children’s mean acceptance for various food items based on children’s ND-status and parents’ fussy eating status.

As can be seen in Figure 1, there was no significant interaction between parents’ fussy eating and children’s ND-status for the children’s mean acceptance scores on any of the food indices: Fruit index: *F*(1, 93) = 1.03, *p* = 0.313, partial η^2^ = 0.01. Vegetable, kale, and salads index: *F*(1, 93) = 0.02, *p* = 0.881, partial η^2^ = 0.00. Nuts and dried fruit index: *F*(1, 82) = 0.20, *p* = 0.653, partial η^2^ = 0.00. Seeds index: *F* (1, 82) = 0.04, *p* = 0.847, partial η^2^ = 0.00.

### 3.2. Children’s Food Consumption (Frequency) Based on Children’s ND-Status and Parents’ Fussy Eating Status

A two-way ANOVA was conducted to examine the effects of parents’ fussy eating and children’s ND-status on children’s mean food consumption (frequency) (Table 4). This score measured how often per week the children consumed various foods. The trend indicated the highest frequency of consumption for foods that might be regarded as healthy i.e., vegetables, fruits and berries, legumes, unprocessed meat, potatoes, water, and milk for children without ND, who had non-fussy eating parents. The lowest frequency of consumption for those foods tended to be for children with ND that had parents who were fussy eaters. The opposite trend was generally seen for foods that might be regarded as unhealthy or processed ham, sausages, forcemeat, French fries, salty crisps/chips or popcorn, cakes, sweets, biscuits, fizzy drinks, and colas where children with ND, and fussy eating parents generally, had the highest frequency of consumption per week.

**Figure 2:**
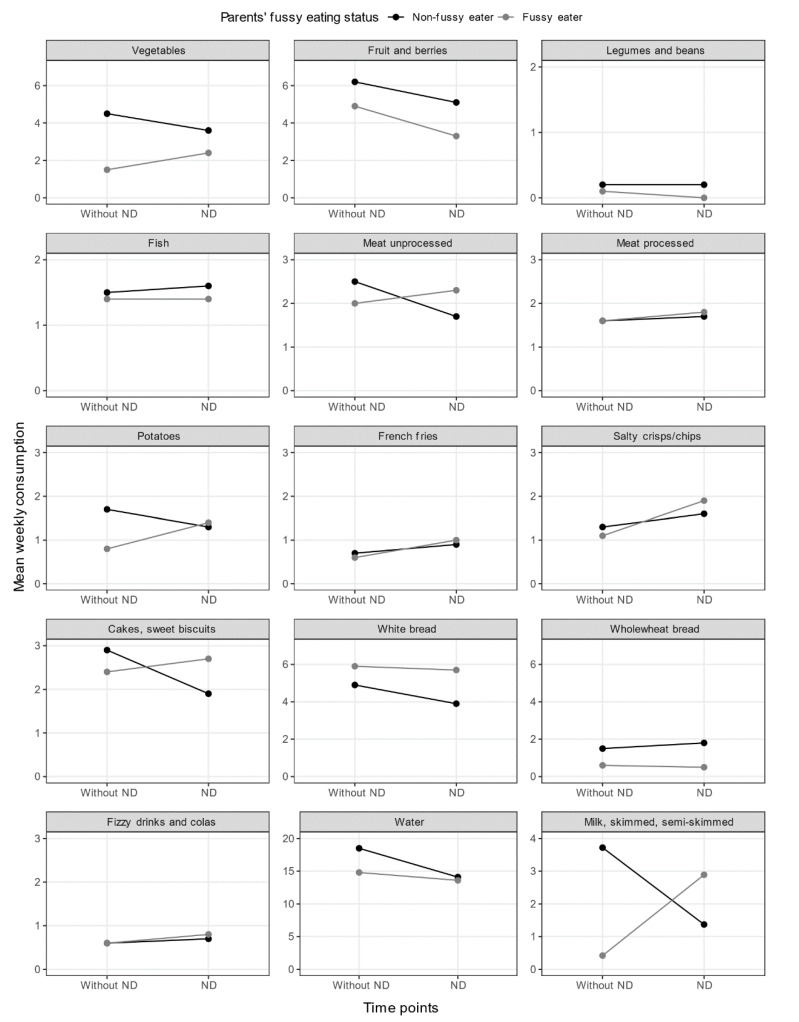
Children’s weekly mean food consumption (frequency) for various food items, based on children’s ND-status, and parents’ fussy eating status.

Despite some of the observed trends (Figure 2), the only significant interaction between parents’ fussy eating status and children’s ND-status for the children’s food frequency scores was for milk, *F*(1, 93) = 7.73, *p* = 0.007, partial η^2^ = 0.08.

The simple main effect of parents’ fussy eating status on children’s mean weekly consumption of milk was significant, *F*(1, 93) = 4.80, *p* = 0.031, partial η^2^ = 0.05. Children without ND who had non-fussy eating parents, consumed milk more frequently than children with ND, a significant mean difference of −2.3, (95% Confidence Interval [CI]: −4.45–−0.22).

The simple main effect of children’s ND-status on their mean weekly consumption of milk was significant, *F*(1, 93) = 7.11, *p* = 0.009, partial η^2^ = 0.07. Children without ND, who had fussy eating parents, consumed milk less frequently on average than children with ND, a significant mean difference of −3.3, (95% CI: −5.76–−0.84).

There was no significant interaction for any of the other foods but trends may be seen for unprocessed meats and cakes/sweet biscuits: vegetables, fresh, frozen, tinned, root vegetables, pulses, *F*(1, 93) = 1.03, *p* = 0.312, partial η^2^ = 0.01; fruit and berries, fresh, frozen, or tinned, *F*(1, 93) = 0.05, *p* = 0.818, partial η^2^ = 0.00; legumes, beans, *F*(1, 93) = 0.12, *p* = 0.733, partial η^2^ = 0.00; fish (not shellfish), *F*(1, 93) = 0.13, *p* = 0.719, partial η^2^ = 0.00; potatoes, cooked and boiled, *F*(1, 93) = 3.35, *p* = 0.071, partial η^2^ = 0.03; unprocessed meat, steaks, minced, *F*(1, 93) = 2.99, *p* = 0.087, partial η^2^ = 0.03; processed meat, ham, sausages, forcemeat, *F*(1, 93) = 0.03, *p* = 0.954, partial η^2^ = 0.00; French fries, fried potatoes, *F*(1, 93) = 0.03, *p* = 0.568, partial η^2^ = 0.00; salty crisps/chips, popcorn, *F*(1, 93) = 1.20, *p* = 0.280, partial η^2^ = 0.13; cakes, sweet biscuits, *F*(1, 93) = 3.67, *p* = 0.058, partial η^2^ = 0.04; white bread, *F*(1, 93) = 0.21, *p* = 0.651, partial η^2^ = 0.00; wholewheat bread, *F*(1, 93) = 0.19, *p* = 0.665, partial η^2^ = 0.00; fizzy drinks, colas, other sugary drinks, *F*(1, 93) = 0.01, *p* = 0.918, partial η^2^ = 0.00; water, *F*(1, 93) = 1.13, *p* = 0.291, partial η^2^ = 0.01.

### 3.3. Children’s Food Related Behaviors Based on Parents’ Fussy Eating

Although there were small and significant effects on some of the food related behavior items before corrections for multiple tests were applied, fussy and non-fussy eating parents did not significantly differ on any of these behaviors after correction. In Table 5, uncorrected items are highlighted where associations were significant before the correction was applied.

For the following categories, there was no significant association between parents’ fussy eating status and children’s eating behaviors: Other eating behaviors; Narrow range; Neophobia, Sensory rejection: texture; Sensory rejection: appearance.

Sensory rejection: taste; There was a significant association between parents’ fussy eating status and children’s eating behavior where a significantly higher proportion of fussy eating parents had children who almost always rejected sour tasting foods. There was no association between parents’ fussy eating status and rejection of foods based on bitter, sweet, or salty foods.

Contact or mixing; There was a significant association between fussy eating parents and children who almost always rejected foods that were mixed or combined (e.g., tuna salad), had “things” in them (e.g., cookie with raisins in it), and refusing foods with sauces on them (e.g., pasta with tomato sauce). There was a significantly higher proportion of children who rejected these foods if they had fussy eating parents than if their parents were non-fussy eaters.

Ritualization/repetition; There was a significant association between fussy eating parents and children who ate the same meal or lunch every day or most days. There was a significantly higher proportion of children who upheld this ritual who had fussy eating parents than had non-fussy eating parents.

Interest in food/social eating; There was a significant association between fussy-eating parents and children who looked forward a lot to eating and had children that missed meals because of being preoccupied or busy and forgetting to eat. There was a higher proportion of children who had non-fussy eating parents, than fussy eating parents that looked forward a lot to eating. Conversely, a higher proportion of children who had fussy eating parents missed meals because of being preoccupied or busy and forgetting to eat, than children with non-fussy eating parents.

## 4. Discussion

The purpose of this study was to analyze the association between parents and their children’s fussy eating. The primary aim was to investigate fussy eating and dietary behaviors, as well as food acceptance and frequency, in a sample of children with and without ND, and the associations with their parents’ fussy eating.

Overall, children with ND accepted fewer food items and consumed unhealthier foods more frequently than children without ND. The same trend was seen for fussy eating parents versus non-fussy eating parents, in which fussy eating parents had children who accepted fewer food items and consumed unhealthier foods more frequently than children whose parents were not fussy eaters. A higher proportion of fussy eating parents than non-fussy eating parents had children who had difficulties with combined foods and hidden ingredients.

### 4.1. Food Acceptance

There was no significant interaction between parents’ fussy eating status and children’s ND-status for the children’s mean acceptance on any of the food indices: Fruit index, Vegetable, kale and salads index, Nuts and dried fruit index, and Seeds index. Results indicated lower mean acceptance scores for children with ND than children without ND on all indices, although not significantly. These results are mostly consistent with studies on children with ND having higher levels of fussy eating than children without ND [26,35,36]. Similarly, children who had fussy-eating parents generally accepted a lower number of foods than non-fussy eating parents. This is in line with reports published on the parental influence on children’s food preferences [9,44], however, no published research is available on the associations between children with and without ND, and fussy eating parents, which makes direct comparisons difficult, as does the lack of significant interactions in our study.

### 4.2. Food Frequency

The only significant interaction between parents’ fussy eating status and children’s ND-status for the children’s food frequency scores was for milk. Children without ND, who had non-fussy eating parents, consumed milk significantly more often than children with ND. Also, children without ND who had fussy eating parents consumed milk significantly less often than children with ND. This interaction effect was somewhat surprising. There seems to be no obvious explanation in the literature. The difference seems to be primarily between children without ND based on their parents’ fussy eating status, which would be interesting to study further. There was no significant interaction for any of the other foods.

The results indicated highest mean frequency of weekly consumption for foods that might be regarded as healthy i.e., vegetables, fruits and berries, legumes, unprocessed meat, potatoes, and water for children without ND who had non-fussy eating parents. Conversely, the lowest mean weekly frequency of consumption for those foods was seen among children with ND when parents were fussy eaters. The opposite was generally seen for foods that might be regarded as bland, salty, sweet, or processed and less healthy ham, sausages, forcemeat, French fries, salty crisps/chips or popcorn, cakes, sweets, biscuits, fizzy drinks, and colas where children with ND and fussy eating parents generally had the highest mean weekly consumption. These findings are consistent with results from other studies showing that children with ND generally consume fewer fruit and vegetables than children without ND [52,53]. The results are also consistent with findings where children with ND had unhealthier diets consisting of sweet and bland foods in terms of texture and taste [26,35,36,54,55,56]. The trend of fussy eating parents, and their lower consumption of healthier foods than for non-fussy eating parents, might be indicative of the parents’ influence on children’s food consumption, as some studies have pointed to fussy eating mothers adversely influencing their children’s food choices [44,45,57]. However, surprisingly, these results did not seem indicative of the specialized diets sometimes applied to children with ASD [27,28], at least not in terms of eliminating dairy or gluten. We also briefly investigated the differences between children with ADHD and ASD, separately, in terms of dairy and gluten, but none were significantly different.

### 4.3. Food-Related Behaviour

The results for children’s food related behaviors and parents’ fussy eating showed that children of fussy eating parents had an overall higher prevalence of fussy eating behaviors. For example, a significantly higher proportion of children who had fussy eating parents rejected foods that were mixed our combined (e.g., tuna salad), had hidden ingredients (e.g., cookie with raisins), or were served with sauce (e.g., pasta with tomato sauce). This is in accordance with research showing children with fussy eating preferring foods that are not mixed or with hidden ingredients [31,32]. The children in our study also seemed to have less interest in food if their parents were fussy eaters i.e., forgot to eat, and did not look forward to meals. This is in line with previous findings which found that parents’ fussy eating may be a strong determinant in their children’s food related behavior [42,58,59,60]. This relationship may be reciprocal, especially for families of children with ND [9,17,20], although we did not measure it for this instance. Several other children’s food-related behaviors in our analyses did not reveal any significant differences based on parents’ fussy eating status.

### 4.4. Strength and Limitations

To the authors’ knowledge, this was one of the first studies to compare children’s fussy eating to their parents’ fussy eating in a sample of children with and without ND. There was an almost equal proportion of children with and without ND, which made comparisons easier when analyzing associations between these groups.

Generally, schools in Iceland are inclusive and the sample was representative of 8–12-year-old children in mainstream schooling.

Participants in the study were self-recruited and were not randomized. Participants consisted mainly of mothers, highly educated and married. Therefore, the study may not be representative of fussy eating children, or their parents, in general. The study was advertised as inclusive in nature, although those with higher social economic status (SES) applied, as is often the case. Using these measures as a proxy for SES the results do not represent families where fruit and vegetables consumption is very low and may not replicate in lower SES context. Otherwise, the participants reflect the Icelandic population. It should be noted that participation was completely free of charge but families may have had to take time off from work or other obligations to be able to attend, causing indirect cost.

For this study, parents, mainly mothers answered all questionnaires. Studies have shown that parents tend to underestimate their children’s picky eating [61] or project their own behaviors onto their children, [41] which may limit the generalizability of the study. However, since the children were rather young, they would not have been able to accurately recollect their food consumption and food-related behavior.

Acceptance and consumption of plant-based foods is a main target for the on-going intervention study that the dataset is derived from. Thus, we have an extensive questionnaire on consumption of items used there. To simplify results, we used four indices; Fruit index; Vegetables, kale, and salads index; Nuts and dried fruits index; and Seeds index. The reason for combining nuts and dried fruits but keeping seeds separate is based on common consumption manners, i.e., nuts and dried fruit are often consumed and sold in combination and are popular as finger foods. Seeds are more prevalent as part of other foods, for instance in baked products, and they have an impact on the appearance of the foods which in turn may affect the acceptance—especially among children with ND.

In this study, we did not have insight into the parents’ own food consumption (acceptance or frequency). As studies show that parental fruit and vegetables consumption may be the strongest predictor of their children’s intake [46], it would have been useful to have information about parents’ foods consumption for comparison.

Even though the Taste Education program itself was a longitudinal study, [62] this paper was centered on the study’s screening data at one point in time and was therefore cross-sectional, with no measures of temporal relationships.

Finally, the increase in familywise error across the reported statistical analyses (two-way ANOVAs and chi-square analyses) was not controlled. Overall, we decided to report unadjusted alphas [50,51] as we consider this analysis of the screening data relatively preliminary, not meant for intervention purposes, and we do encourage replication with larger participant samples.

## 5. Conclusions

The findings from this study highlight the need for further investigation into the relationships between parents’ influence on their children’s eating behavior and food consumption, as well as possible reciprocal impacts. This is especially important since there are no prior published studies on the associations of parents’ fussy eating with children’s ND-status. It is possible that parents’ fussy eating may affect children with ND differently to children without ND, although parents’ eating behaviors need to be investigated further. Findings of this current study may be helpful as a step towards improving parental awareness on their own food consumption and eating behaviors and how they may influence their children’s eating behaviors.

## Figures and Tables

**Table 1:** The 14 statements used to divide parents into two groups based on parents’ fussy eating status (fussy or not). ^ǂ^

Statements
4. I am a fussy eater;
5. My food choices are very monotonous;
8. I do not like to try new foods;
9. I reject bitter foods;
10. I reject sour foods;
11. I reject sweet foods;
12. I reject salty foods;
13. I avoid foods with certain texture;
14. I avoid foods which have a slippery texture;
17. I reject foods that are mixed or combined;
18. I reject food that has lumps in it;
19. I reject food that has “bits” in it;
21. I reject foods if I cannot see the ingredients with my own eyes;
22. I try not to let different types of foods touch on my plate.

^ǂ^ Items numbers correspond with the Adult Picky Eating Questionnaire [48].

**Table 2:** Characteristics of parents and children in the study. Results are presented as n (%).

Children’s Characteristics, *n* (%)		*n* = 95
Sex	Female	54 (56.8)
	Male	43 (45.2)
ND-status	Without ND	47 (49.5)
	With ND	48 (50.5)
**Parents’ characteristics, *n* (%)**		n = 97 ^ǂ^
Sex	Female	92 (94.8)
	Male	5 (5.2)
Educational level	No higher education	6 (6.2)
	Vocational education	17 (17.5)
	University level	74 (76.3)
Occupational status	Full-time occupation	73 (75.3)
	Part-time occupation	11 (11.3)
	Student	10 (10.3)
	Other	11 (11.3)
Marital status	Single	14 (14.4)
	Divorced	8 (8.2)
	Married	75 (77.3)
Number of children in the household	1 child	13 (13.4)
	2 children	43 (44.3)
	3 children	30 (30.9)
	4 or more children	11 (11.3)
Fussy eating status	Fussy eater	37 (38.1)
	Not a fussy eater	60 (61.9)

^ǂ^ Two children had both parents fill out screening questionnaires.

**Table 3:** Children’s food acceptance scores (number of food items accepted) for all food indices based on children’s ND-status, and parents’ fussy eating status. Results are presented as Mean (SD).

Children’s Food Consumption (Number of Food Items Accepted), Mean (SD)	Children’s ND-Status	Total Sample (*n* = 97)	Parent Is a Fussy Eater (*n* = 37)	Parent Is Not a Fussy Eater (*n* = 60)
Fruit index	Without ND	8.0 (3.7)	7.3 (3.3)	8.4 (4.0)
	With ND	6.9 (3.7)	6.6 (2.9)	7.0 (4.1)
Vegetable, kale, and salad index	Without ND	7.1 (5.7)	5.4 (4.2)	8.1 (6.3)
	With ND	6.8 (5.7)	5.4 (4.5)	7.6 (6.4)
Nuts and dried fruit index	Without ND	3.2 (2.6)	2.8 (2.5)	3.5 (2.6)
	With ND	2.8 (2.5)	2.6 (2.1)	2.9 (2.7)
Seeds index	Without ND	0.9 (1.4)	0.7 (1.3)	1.0 (1.5)
	With ND	0.9 (1.6)	0.9 (1.2)	1.0 (1.6)

**Table 4:** Children’s mean food consumption (frequency) for various food items, based on children’s ND-status, and parents’ fussy eating status. Results are presented as Mean (SD).

Children‘s Food Consumption (Frequency), Mean (SD)	Children‘s ND-Status	Total Sample (*n*=97)	Parent Is a Fussy Eater (*n* = 37)	Parent Is Not a Fussy Eater (*n* = 60)
Vegetables; fresh, frozen, tinned, root vegetables, pulses	Without ND	3.4 (4.7)	1.5 (2.0)	4.5 (5.4)
	With ND	3.1 (3.6)	2.4 (2.3)	3.6 (4.2)
Fruit and berries; fresh, frozen, or tinned	Without ND	5.7 (5.3)	4.9 (4.9)	6.2 (5.5)
	With ND	4.4 (5.3)	3.3 (4.8)	5.1 (5.5)
Legumes, beans	Without ND	0.2 (0.4)	0.1 (0.2)	0.2 (0.5)
	With ND	0.1 (0.6)	0.0 (0.1)	0.2 (0.8)
Fish (not shellfish)	Without ND	1.5 (1.1)	1.4 (1.3)	1.5 (1.3)
	With ND	1.5 (1.2)	1.4 (0.9)	1.6 (2.7)
Meat, unprocessed; steaks, minced	Without ND	2.3 (1.4)	2.0 (0.3)	2.5 (0.3)
	With ND	1.9 (1.5)	2.3 (1.3)	1.7 (1.6)
Meat, processed; ham, sausages, forcemeat	Without ND	1.6 (1.8)	1.6 (1.7)	1.6 (1.8)
	With ND	1.7 (1.6)	1.8 (1.5)	1.7 (1.6)
Potatoes; baked, boiled	Without ND	1.4 (1.4)	0.8 (0.9)	1.7 (1.6)
	With ND	1.3 (1.1)	1.4 (1.1)	1.3 (1.2)
French fries, fried potatoes	Without ND	0.7 (0.5)	0.6 (0.6)	0.7 (0.4)
	With ND	1.0 (0.8)	1.0 (1.0)	0.9 (0.7)
Salty crisps/chips, popcorn	Without ND	1.2 (0.9)	1.1 (0.8)	1.3 (1.0)
	With ND	1.7 (1.1)	1.9 (1.3)	1.6 (1.0)
Cakes, sweet biscuits	Without ND	2.7 (2.0)	2.4 (1.9)	2.9 (2.1)
	With ND	2.2 (1.5)	2.7 (1.6)	1.9 (1.3)
White bread	Without ND	5.3 (4.4)	5.9 (5.4)	4.9 (3.6)
	With ND	4.6 (4.6)	5.7 (4.8)	3.9 (4.4)
Wholewheat bread	Without ND	1.2 (1.8)	0.6 (1.2)	1.5 (2.0)
	With ND	1.3 (2.0)	0.5 (0.9)	1.8 (2.3)
Fizzy drinks; colas and other sugary drinks	Without ND	0.6 (0.8)	0.6 (1.0)	0.6 (0.6)
	With ND	0.7 (1.3)	0.8 (1.2)	0.7 (1.3)
Water	Without ND	17.1 (7.0)	14.8 (8.4)	18.5 (5.6)
	With ND	13.9 (7.6)	13.6 (7.5)	14.1 (7.7)
Milk; skimmed, semi-skimmed	Without ND	2.5 (4.7)	0.4 (1.1)	3.7 (5.5)
	With ND	2.0 (3.9)	2.9 (5.1)	1.4 (2.8)

**Table 5:** Children’s food related behaviors based on parents’ fussy eating. Results are presented as n (%). ^ǂ^

Children‘s Food Consumption (Frequency), Mean (SD)	Total Sample (*n* = 97)	Parent Is a Fussy Eater (*n* = 37)	Parent Is Not a Fussy Eater (*n* = 60)	*χ*^2^ (1)	*φ*	*p*
**Other eating behaviors**						
1. My child has food allergies	10 (10.3)	1 (2.7)	9 (15.0)	3.74	−0.20	0.053
**Narrow range**						
2. My child eats from a very narrow range of foods	48 (49.5)	20 (54.1)	28 (46.7)	0.50	0.07	0.480
3. My child almost always avoids one or more major food groups	64 (66.0)	27 (73.0)	37 (61.7)	1.30	0.12	0.254
**Neophobia**						
4. My child does not like to try new foods	82 (84.5)	31 (83.8)	51 (85.0)	0.03	−0.02	0.872
**Sensory rejection: taste**						
5. My child almost always rejects bitter foods	71 (73.2)	31 (83.8)	40 (66.7)	3.42	0.19	0.064
6. My child almost always rejects sour foods	48 (49.5)	23 (62.2)	25 (41.7)	3.85	0.20	0.050
7. My child almost always rejects sweet foods	5 (5.2)	3 (8.1)	2 (3.3)	1.07	0.10	0.302
8. My child almost always rejects salty foods	11 (11.3)	6 (16.2)	5 (8.3)	1.41	0.12	0.234
**Sensory rejection: texture**						
9. My child almost always avoids foods with a particular texture (crunchy, gelatinous, or very chewy)	58 (60.4)	26 (70.3)	32 (54.2)	2.40	0.16	0.118
10. My child almost always avoids foods that are slippery or slimy	60 (61.9)	25 (67.6)	35 (58.3)	0.83	0.09	0.363
**Sensory rejection: appearance**						
11. My child almost always rejects foods that are a particular color	5 (5.2)	2 (5.4)	3 (5.0)	0.01	0.01	0.930
12. My child almost always prefers to eat only foods that are a particular color	1 (1.0)	0 (0.0)	1 (1.7)	0.62	−0.08	0.430
**Contact or mixing**						
13. My child almost always rejects foods that are mixed or combined (e.g., tuna salad)	57 (60.0)	29 (78.4)	28 (48.3)	8.53	0.30	0.003
14. My child almost always rejects foods with “lumps” in them (e.g., a stew)	1 (62.9)	27 (73.0)	34 (56.7)	2.61	0.16	0.106
15. My child almost always refuses foods that have “things” in them (e.g., cookie with raisons in it)	40 (41.2)	20 (54.1)	20 (33.3)	4.05	0.20	0.044
16. My child almost always refuses foods with sauces on them (e.g., pasta with tomato sauce)	41 (42.3)	21 (56.8)	20 (33.3)	4.86	0.22	0.028
17. My child almost always rejects foods if there is something they cannot see in them (e.g., filled foods like eggroll or ravioli)	41 (58.3)	26 (70.3)	30 (50.8)	3.53	0.19	0.060
18. My child tries to not let different types of foods touch on their plate	53 (54.6)	24 (64.9)	29 (48.3)	2.52	0.16	0.112
19. My child almost always rejects foods that have touched on their plate	27 (27.8)	12 (32.4)	15 (25.0)	0.63	0.08	0.428
**Ritualization/repetition**						
20. My child almost always prefers to eat with a special persons(s), in a special place or with special utensils/dishes	10 (10.3)	5 (13.5)	5 (8.3)	0.66	0.08	0.415
21. My child usually eats foods in sequence in the main course	27 (27.8)	14 (37.8)	13 (21.7)	2.98	0.17	0.084
22. My child often eats foods in an unusual order	7 (7.2)	5 (13.5)	2 (3.3)	3.54	0.19	0.060
23. My child eats the same meal for breakfast every day or most days	79 (81.4)	32 (86.5)	47 (78.3)	1.01	0.10	0.316
24. My child eats the same meal for lunch every day or most days	17 (17.5)	11 (29.7)	6 (10.0)	6.16	0.25	0.013
25. My child eats the same meal for supper every day or most days	15 (15.5)	9 (24.3)	6 (10.0)	3.59	0.19	0.058
26. My child usually does not want to eat foods if they have seen someone else touch it	27 (27.8)	11 (29.7)	16 (26.7)	0.11	0.03	0.744
**Interest in food/social eating**						
27. My child looks forward a lot to eating	18 (19.1)	2 (5.7)	16 (27.1)	6.50	−0.26	0.011
28. My child often misses meals because of being preoccupied or busy and forgets to eat	50 (51.5)	24 (64.9)	26 (43.3)	4.25	0.21	0.039
29. My child prefers to leave a clean plate	14 (14.6)	3 (8.3)	11 (18.3)	1.81	−0.14	0.179
30. When my child is invited to dinner, they worry that there may be nothing that they can eat	23 (23.7)	6 (16.2)	17 (28.3)	1.86	−0.14	0.173

^ǂ^ Alphas are unadjusted.

## Data Availability

Data is available: The datasets generated during and/or analyzed during the current study are available from the corresponding author on reasonable request.
